# Effectiveness and Safety of Kangjia Decoction Granules for the Treatment of Hashimoto Thyroiditis: Protocol for a Randomized, Double-Blinded, Placebo-Controlled, Multicenter Clinical Trial

**DOI:** 10.2196/80993

**Published:** 2026-01-26

**Authors:** Duanchun Zhang, Dan Zhang, Xiaoxiao Qu, Huihong Cao, Yanming He

**Affiliations:** 1 Department of Endocrinology Yueyang Hospital of Integrated Traditional Chinese and Western Medicine Shanghai University of Traditional Chinese Medicine Shanghai China; 2 Department of Endocrinology Shanghai Minhang of Integrated Traditional Chinese and Western Medicine Shanghai China; 3 Department of Critical Care Medicine Shuguang Hospital Shanghai University of Traditional Chinese Medicine Shanghai China

**Keywords:** kangjia decoction granules, Hashimoto thyroiditis, evidence-based clinical trial, randomized controlled trial, protocol

## Abstract

**Background:**

Hashimoto thyroiditis (HT) is a chronic inflammation of the thyroid gland mediated by autoimmune disorders, often leading to hypothyroidism and a significant reduction in a patient’s quality of life. At the time of this writing, there is a lack of effective clinical treatments for early-stage HT. Kangjia decoction granules (KDGs) were developed based on clinical experience and results analysis, showing promising outcomes in improving antibody levels and quality of life in patients with HT. However, there is a lack of further evaluation of the efficacy and safety of KDGs.

**Objective:**

This pilot study aims to further understand and validate the efficacy and safety of KDGs for treating HT through clinical research and comprehensively assess the benefits of this intervention for patients.

**Methods:**

This study is a multicenter, randomized, double-blind, placebo-controlled clinical trial. Participants meeting the HT diagnostic criteria will be randomly allocated to the intervention and control groups (n1=n2=70). The intervention group will receive KDG treatment, whereas the control group will receive a placebo treatment. All participants will undergo treatment for 3 months. Changes in antithyroid peroxidase antibody (TPOAb) levels will be the primary outcome. Secondary outcomes include antithyroglobulin antibodies (TGAb), thyrotropin, also known as thyroid stimulating hormone (TSH), triiodothyronine (T3), thyroid hormone (T4), serum free triiodothyronine (FT3), serum free thyroxine (FT4), thyroid ultrasonography, *IL17* mRNA and *FOXP3* mRNA, traditional Chinese medicine (TCM) syndrome efficacy scores, and quality of life scale scores. Throughout the treatment and follow-up periods, safety indicators, such as routine blood and urine tests, hepatic and renal function, electrocardiography, and major adverse reactions, will be monitored.

**Results:**

The research protocol and informed consent form received approval from the Clinical Research Ethics Committee of Yueyang Hospital of Integrated Traditional Chinese and Western Medicine, affiliated with Shanghai University of Traditional Chinese Medicine, on December 14, 2022 (Approval No. 2022-123). Participant recruitment commenced in June 2023. All intervention and concurrent data collection activities were scheduled for completion by October 2025. Data management is still ongoing; therefore, data analysis has not yet been performed.

**Conclusions:**

This study’s findings will offer initial clinical evidence regarding the efficacy of the TCM compound KDGs in modulating peripheral immunity in patients with HT, decreasing autoimmune antibody levels, ameliorating TCM syndromes, and enhancing quality of life. These results will serve as a basis for future large-scale trial designs.

**Trial Registration:**

China Clinical Trials Registry ChiCTR2300070184; https://www.chictr.org.cn/showprojEN.html?proj=189169

**International Registered Report Identifier (IRRID):**

DERR1-10.2196/80993

## Introduction

Hashimoto thyroiditis (HT), also known as chronic lymphocytic thyroiditis, is a chronic thyroid inflammation mediated by autoimmune disorders [1]. The prevalence of HT in the female population worldwide currently exceeds 10% and is increasing annually, showing a trend toward younger ages [2]. Patients with HT experience diffuse thyroid changes and have elevated levels of thyroid peroxidase antibodies (TPOAb) and thyroglobulin antibodies (TGAb) in the serum [3,4]. The levels of these antibodies to some extent reflect the severity of HT and serve as important indicators for evaluating its occurrence, development, and prognosis. In the early stages, patients with HT may show no obvious symptoms or may experience discomfort or swelling in the neck. As the disease progresses, some patients may exhibit hyperthyroidism symptoms, such as palpitations, sweating, and irritability. In later stages, the disease can lead to hypothyroidism, manifesting as delayed reactions, impaired memory, and other symptoms that significantly impact the quality of life. Studies have also demonstrated a close association between this disease and papillary thyroid cancer, as well as reproductive health issues, such as premature birth and fetal deformities. Therefore, HT is receiving increasing attention globally [5-7].

The pathogenesis of HT remains unclear. Most studies suggest that its onset is due to the disruption of the local immune balance in the thyroid gland, leading to the body’s mistaken recognition of its own thyroid cell structure as an antigen, triggering the production of antibodies and initiating the formation of antigen–antibody complexes that lead to inflammation. Recent research has indicated that an imbalance between helper T cell 17 (Th17) and regulatory T cells (Tregs) is closely associated with the occurrence and development of HT [8,9]. Th17 cells mediate inflammation, whereas Tregs mediate immune suppression. Th17 and Treg cells work together to maintain a balance in the immune microenvironment of the body. During the progression of HT, Th17 cells increase in number, and their secretion of cytokines, such as interleukin (IL)-17, is elevated, whereas the number of Tregs and their secretion of FOXP3 are relatively reduced [10]. As HT advances, an imbalanced immune response and dysregulated recognition processes initiate apoptosis, leading to increased follicular cell apoptosis, irreversible fibrosis of follicular structures, reduced effective synthesis of thyroid hormone cells, and, ultimately, hypothyroidism [11]. At this stage, most patients require lifelong thyroid hormone replacement. Even when thyroid hormone levels are restored to normal, patients often experience symptoms such as fatigue, lower limb edema, low mood, and anxiety, which severely affect their quality of life [12,13].

A current clinical shortcoming is the lack of treatment options for patients with early-stage HT, especially for immune dysregulation-related causes. Treatment is typically initiated only when patients develop hypothyroidism, hyperthyroidism, or other complications when levothyroxine replacement or symptomatic treatment is provided. There is, therefore, an urgent need in clinical practice for treatment approaches that target overall immune regulation as the treatment goal, not only to address the disease itself but also to improve the overall quality of life of patients.

Recent research has shown that traditional Chinese medicine (TCM) plays an important role in the treatment of HT through its multitarget actions [14]. The research team integrated, analyzed, and statistically processed clinical data from hundreds of patients in the early stages of the disease and found that certain combinations of Chinese herbal medicines effectively reduced TPOAb and TGAb levels in patients with HT and significantly improved their clinical symptoms. Based on clinical experience and result analysis, the research team formulated the kangjia decoction granules (KDGs; Astragali Radix 18 g, Prunella vulgaris 15 g, Atractylodis Macrocephalae Rhizoma 6 g, Forsythiae Fructus 6 g, Rehmannia 6 g, and Cyperus Rotundus 6 g) for the treatment of HT.

This randomized, parallel, placebo-controlled, double-blind, and multicenter clinical trial aims to evaluate the effectiveness and safety of the KDGs and confirm their ability to regulate the immune system, treat HT, delay or prevent the occurrence of hypothyroidism, and improve quality of life.

## Methods

### Study Design

This is a randomized, double-blind, placebo-controlled, multicenter clinical trial in which the participants are randomized in a 1:1 ratio to either the treatment group (KDGs group) or the control group (placebo group). Each group will undergo a 12-week treatment. See Multimedia Appendix 1 for the SPIRIT (Standard Protocol Items: Recommendations for Interventional Trials) 2025 checklist [15].

We will record all baseline observations during the screening period. Throughout the treatment period, participants will have monthly follow-up visits and complete TCM evidence-based efficacy and quality of life scale score assessments. TPOAb, TGAb, thyroid stimulating hormone (TSH), triiodothyronine (T3), thyroid hormone (T4), serum free triiodothyronine (FT3), serum free thyroxine (FT4), thyroid ultrasonography, and *IL17* mRNA and FOXP3 mRNA levels will be examined and recorded before and after treatment. The schedule of participants is shown in Table 1.

**Table 1:** Study schedule of assessments.

Item	Baseline (day 7~0)	0 weeks (day 0; visit 1)	4 weeks (day 28; visit 2)	8 weeks (day 56; visit 3)	12 weeks (day 84; visit 4)
Informed consent	✓				
Inclusion/exclusion criteria	✓				
Demographic information	✓				
History taking and recording	✓	✓	✓	✓	✓
Physical examination	✓	✓	✓	✓	✓
Traditional Chinese medicine evidence-based efficacy score		✓	✓	✓	✓
Quality of life scale score		✓	✓	✓	✓
Electrocardiogram	✓				✓
Blood test	✓				✓
Urinalysis	✓				✓
Liver function	✓				✓
Kidney function	✓				✓
TPOAb^a^	✓				✓
TGAb^b^	✓				✓
TSH^c^	✓				✓
T3^d^	✓				✓
T4^e^	✓				✓
FT3^f^	✓				✓
FT4^g^	✓				✓
IL^h^-17/FOXP3	✓				✓×
Thyroid ultrasound	✓				✓
Record of study drug usage	✓	✓	✓	✓	✓
Adverse event record	When necessary	When necessary	When necessary	When necessary	When necessary
Record of combined medication use	✓	✓	✓	✓	✓
Analysis of causes of shedding	✓	✓	✓	✓	✓

^a^TPOAb: thyroid peroxidase antibody.

^b^TGAb: thyroglobulin antibodies.

^c^TSH: thyroid stimulating hormone.

^d^T3: triiodothyronine.

^e^T4: thyroid hormone.

^f^FT3: free triiodothyronine.

^g^FT4: free thyroxine.

^h^IL: interleukin.

### Participant Screening and Selection

Per the 2018 China Guidelines for Diagnosis and Treatment of Thyroid Diseases [16], HT is identified by a diffuse, firm goiter, often with isthmus or pyramidal lobe enlargement, and positive serum TPOAb or TGAb. Diagnosis can also be confirmed through fine needle aspiration cytology. Outpatients or inpatients meeting the diagnostic criteria for HT will be assessed for inclusion and exclusion (Textbox 1). Patients will not be recruited if they do not meet any of the inclusion criteria or meet any of the refusal or termination criteria. Participants are required to provide written informed consent, cooperate with the physician’s treatment and follow-up, and provide information within the scope of ethical approval. Patients who do not meet these requirements will be excluded from the study. During this period, the participant’s medical history, physical examinations, blood samples, and medical records will be collected and preserved.

**Inclusion, exclusion, and rejection criteria and termination.**

**Inclusion criteria:**

1. Patients meet the diagnostic criteria of Hashimoto thyroiditis based on the guidelines for the diagnosis and treatment of thyroid diseases in China;

2. The Traditional Chinese medicine dialectical belongs to the syndrome of liver depression and spleen deficiency;

3. Age ≥ 18 and ≤ 65 years; gender not restricted;

4. Triiodothyronine, thyroid hormone, free triiodothyronine, and free thyroxine in the normal range of the tested values, thyroid stimulating hormone<10 mIU/L;

5. Aspartate transaminase (AST) and alanine transaminase (ALT) ≤ 3 × ULN creatinine ≤ 1.5 ULN;

6. The patients understand and agree to participate in this study and voluntarily sign the informed consent.

**Exclusion criteria:**

1. Patients with poorly controlled blood pressure on medication, systolic blood pressure160 mm Hg, or diastolic blood pressure>100 mm Hg;

2. Patients who have endured or are enduring hyperthyroidism or hypothyroidism or patients with hormone supplementation therapy after thyroid tumor surgery;

3. Patients who had myocardial infarction, heart failure, or unstable angina within six months prior to enrollment;

4. Patients with a history of stroke within 6 months prior to enrollment;

5. Pregnant or lactating women;

6. Patients with chronic alcoholism, drug dependence, or mental illness;

7. Patients with a history of tumors;

8. Patients with known hypersensitivity to the test drug or components thereof;

9. Participation or ongoing participation in other interventional clinical studies within one month prior to enrollment;

10. Those who, in the opinion of the investigator, should not participate in this clinical study.

**Criteria for strike-out, discontinuance, and shedding:**

1. Strike-out criteria

1.1 Misscheduling and misinclusion;

1.2 Those who, in the opinion of the investigator, are not suitable for continued participation in this study.

2. Discontinuation criteria

2.1 Adverse events or serious adverse events occurring during the trial, making the participant unsuitable for continued participation in the study;

2.2 Serious deterioration of the patient’s disease or the occurrence of certain comorbidities, complications, and special physiological changes, leading to unsuitability for continued participation in the study.

3. Desertion criteria

3.1 Participants who show poor compliance, which is not in accordance with the provisions of the treatment or the follow-up investigation;

3.2 Existence of incomplete medical records, affecting the evaluation of efficacy;

3.3 Participants withdraw on their own.

The study aims to recruit 140 participants across 3 units. These 3 units include primary coordinating and implementing units. Yueyang Hospital of Integrated Traditional Chinese and Western Medicine, affiliated with Shanghai University of Traditional Chinese Medicine, will recruit 80 participants. Longhua Hospital and Putuo Hospital, both affiliated with the Shanghai University of Traditional Chinese Medicine, will each recruit 30 participants. Each unit is responsible for specific aspects of the study, including patient recruitment, specimen collection, and data collection. Imaging examinations will be conducted in the department of each unit. Laboratory testing and data analysis will be overseen by the Clinical Research Center of Yueyang Hospital.

To ensure participant safety, blinding, data quality, and compliance with the research protocol, all personnel directly involved in this study will receive comprehensive training and adhere to the Good Clinical Practice (GCP) guidelines.

### Blinding

Blinding preservation: a two-tier blinding approach was implemented, with the first tier involving the assignment of group codes corresponding to each drug number (Group A or Group B), and the second tier involving the concealment of the actual drug types for the two groups. Sealed blinding documentation was duplicated and entrusted to the clinical research unit for safekeeping.

Blinding procedures: this study used a double-blinded method. Both blinding processes were overseen by the designated personnel responsible for maintaining the blinding. The first blinding involved the specification of Groups A and B, whereas the second blinding involved specifying the test and control groups. Blinding procedures were overseen by the project leader of the clinical study unit, the principal investigator, and the statistician. Randomization order not available to participant registrars and intervention assigners

Emergency blinding: emergency blinding was permitted in the event of serious complications or adverse events during the study that could impact conduct and treatment decisions. In such cases, the researcher, project leader, and clinical supervisor were required to be present, and detailed records of the reason, time, and location of blinding were documented and signed. All data were preserved.

### Sample Size Calculation

This study was based on a clinical trial of a Chinese herbal medicine compound KDGs, for the treatment of HT. The study followed a randomized, double-blind, placebo-controlled design, with a change in the TPOAb level after 12 weeks of treatment serving as the primary efficacy endpoint. Using a 1:1 allocation ratio, the study participants were randomly assigned to either the TCM (KDGs) or the placebo group (placebo granules).

To determine the sample size, we used a clinical study sample size calculation formula:



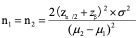



Considering the mean change in TPOAb levels before and after treatment in the treatment group (μ1=120, SD σ1=200) and the control group (μ2=20, SD σ2=200) based on previous study results [17], the study aimed to achieve a power of 80% (1 - β=.80), with a significance level of 0.05 (two-sided) and a β of .20. The calculated sample size was 64, and with a 10% dropout rate, 140 participants were required for the study, with 70 individuals in each group.

### Interventions

#### The KDGs Group

One packet of KDGs (Astragali Radix 18 g, Prunella vulgaris 15 g, Atractylodis Macrocephalae Rhizoma 6 g, Forsythiae Fructus 6 g, Rehmannia 6 g, and Cyperus Rotundus 6 g) is to be taken twice daily, mixed with warm water. The time of administration is half an hour after the morning and evening meals. The treatment will last for 3 months.

#### Placebo Group

One packet of placebo granules is to be taken twice daily, mixed with warm water. The time of administration is half an hour after the morning and evening meals. The treatment will last for 3 months.

The participants will return any unused pills and bottles during each follow-up visit. Unused pills will be counted and documented in the corresponding case report. KDGs and placebo granules will be provided by Shanghai Chiatai Qingchunbao Co Ltd.

### Investigation Product

#### Manufacturing

The 6 herbal ingredients are to be extracted twice, each time with a 12-fold quantity of water, for 1.5 hours per extraction. The combined extracts are filtered, and the filtrate is concentrated under reduced vacuum to produce a dense extract with a relative density of 1.22-1.29 (at 60 °C). Subsequently, 1 g of sucralose, 4.4 g of caramel flavor essence, and a sufficient quantity of dextrin are added to the dense extract, followed by granulation and drying, to yield a final product of 1000 g.

#### Quality Control

The KDGs are manufactured and controlled according to the Chinese Pharmacopoeia and internal quality standards. The quality assessment methods are fully validated. Each batch, as documented in the Certificate of Analysis (No. 23W-004), must meet all release specifications outlined in the following table before clinical use (Multimedia Appendix 2. Stability data confirm the product remains within specifications under recommended storage conditions throughout the study.

#### Placebo Verification

A placebo similarity assessment was conducted to evaluate the blinding integrity of the investigational product. Using a standardized manual scoring method, KDGs (Batch: 20230401) and its matched placebo (Batch: 20230401) were determined to be highly comparable in terms of clarity, odor, and color. However, the similarity in taste was assessed to be less comparable. This finding has been documented and considered in the overall blinding strategy for the trial Multimedia Appendix 3.

### Laboratory Methods for Biomarker Analysis: Measurement of IL-17 and FOXP3 mRNA Expression

1. Sample processing

Total RNA will be extracted from 250 μL of citrate-anticoagulated peripheral blood using Trizol reagent. Briefly, the lysate will be stored at –80 °C until processing. After thawing, chloroform will be added for phase separation, followed by RNA precipitation with isopropanol. The RNA pellet will be washed with 75% ethanol, air-dried, and finally dissolved in RNase-free water.

1. cDNA synthesis.
2. cDNA was synthesized from total RNA using a commercial reverse transcription kit, following the manufacturer’s instructions. The reaction mix was gently vortexed, centrifuged, and then incubated in a thermal cycler to complete the reverse transcription.
3. Quantitative polymerase chain reaction (PCR)
4. Gene expression was quantified by real-time PCR using SYBR Green chemistry. Reactions were set up in triplicate for each cDNA sample in a total volume of 20 µL, containing diluted cDNA, master mix, and specific primers. Amplification was carried out on a real-time PCR detection system with standard cycling parameters, including a melt curve stage for amplicon validation. For detailed primer sequences, please refer to
  Table 2
  .

**Table 2:** Primer sequence.

Gene	Primer sequence (5'-3')
GAPDH Forward primer (Human）	TGGGCAAGGTCATCCCTGAG
GAPDH Reverse primer (Human）	GCGTCAAAGGTGGAGGAGTG
IL17A Forward primer (Human）	TCCCACGAAATCCAGGATGC
IL17A Reverse primer (Human）	GCACTTTGCCTCCCAGATCA
FOXP3 Forward primer (Human）	TCCAGGACAGGCCACATTTC
FOXP3 Reverse primer (Human）	GCCACGTTGATCCCAGGT

### Data Collection and Management

#### Data Collection

Data will be collected from the participants’ daily symptom records, case report form (CRF) records of the trial group’s medical staff, and patients’ medical records. The data will be collected and managed using the REDCap (Research Electronic Data Capture; Vanderbilt University) electronic data capture tool at the Yueyang Integrated Traditional Chinese and Western Medicine Hospital of Shanghai University of Traditional Chinese Medicine. REDCap is a secure web software platform designed to support research data collection. This platform provides (1) an intuitive interface for data entry validation, (2) audit trails to track data manipulation and export procedures, (3) automated export procedures for seamless data download into popular statistical packages, and (4) procedures for data integration and interoperability with external resources.

#### Data Entry and Data Query Forms

The responsibility for data entry and management lies with the designated data administrator, who will establish the database and validation program. The data will then be input and verified by 2 trained data-entry operators. After reviewing the CRF, the identified input errors will be rectified until the input data matches the CRF. Any queries related to the CRF will be conveyed to the investigator using a data query table. These data query tables must be processed, signed, and dated by the researcher in a timely manner. Resolved data issues must be returned to the data manager, who will adjust and validate the data based on the feedback, update the database accordingly, and reevaluate the necessary data problem table.

#### Database Locking

Once the accuracy of the established database has been confirmed by blind checking, the database will be locked. Thus, the locked data cannot be altered. If any data revisions are deemed necessary after locking, an official statement signed jointly by the applicant, principal investigator, experimental project manager, statistician, and data manager must be provided. Furthermore, revisions will be made to the statistical analyses. The Data and Safety Monitoring Board will consist of an internal medicine physician, a sonographer, a medical statistician, and an ethicist and will conduct assessments throughout the study.

#### Electronic Data Encryption

This study will use REDCap for electronic data encryption. REDCap uses a comprehensive encryption strategy to ensure end-to-end data security. All data transmissions between browsers and the REDCap application are protected by Transport Layer Security/Secure Sockets Layer encryption. Data at rest is stored in a secured network environment with regular backups. For mobile data collection, devices should be password-protected to enable full device encryption. Additionally, application programming interface keys should not be hard-coded in scripts but stored securely using encrypted keyrings, with proper configuration to prevent accidental exposure of credentials in unencrypted files.

#### Participant Compliance

##### Informed Consent

A thorough process must be followed to secure participant understanding and cooperation. The investigational product and required laboratory tests must be provided free of charge.

##### Compliance Monitoring

The medication measurement should be used to monitor adherence. Compliance should be calculated as follows: (actual medication dose administered / planned medication dose) × 100%. Compliance between 80% and 120% is deemed acceptable.

##### Management of Poor Compliance

All concomitant medications in the eCRF should be recorded. Follow-up for participants with poor therapeutic response or compliance must be intensified (<80% or >120%).

### Evaluation

#### Primary Outcome

- Difference in TPOAb levels before and after treatment.

#### Secondary Outcomes

- TGAb, TSH, T3, T4, FT3, FT4, thyroid ultrasound,
  IL17 mRNA,
  FOXP3 mRNA (peripheral blood),
  TCM syndrome score, quality of life scale score.

#### Security Outcome

- Blood routine, urine routine, hepatic and renal function, and electrocardiogram.

#### Adverse Event Observation and Recording

Following entry into the clinical trial, the patient’s vital signs, symptoms, any adverse events related to the disease, and laboratory parameters will be recorded. Thus, the occurrence of adverse events may not necessarily be related to the study drugs. Adverse events were assessed and graded according to the Common Terminology Criteria for Adverse Events (CTCAE) version 5.0. Grade 1 (mild): asymptomatic or mild symptoms; clinical or diagnostic observations only; intervention not indicated. Grade 2 (moderate): minimal, local, or noninvasive intervention indicated; limiting age-appropriate instrumental activities of daily living. Grade 3 (severe or medically significant): medically significant but not immediately life-threatening; hospitalization or prolongation of hospitalization indicated; disabling; limiting self-care activities of daily living. Grade 4 (life-threatening): urgent intervention indicated. Grade 5 (death): death related to the adverse event. The initial report of any serious adverse event must be made within 24 hours of discovery to the principal investigator, Dr He Yanming. The principal investigator is responsible for ensuring the subsequent report is escalated to the Research Administration Office at Yueyang Hospital of Integrated Traditional Chinese and Western Medicine (telephone: 65161782, extension Research Admin). The final mandatory report must be submitted to the Drug Research Supervision Department of the National Medical Products Administration (telephone: 010-68313344-1013). Any causal relationship between the adverse event and the investigational drug will be determined by classification. All serious adverse events were followed until resolution or until the event was considered stable.

In the case of a serious adverse event in a participant, the details of the medication must be disclosed, and unblinding can only be performed with the consent of the principal investigator. Once an emergency letter has been read, the case will be considered a dropout.

#### Evaluation Index Detection Method

Serological analysis was conducted at the laboratory department of Yueyang Hospital of Integrated Traditional Chinese and Western Medicine, affiliated with Shanghai University of Traditional Chinese Medicine. Blood-related parameters were assessed using a Roche automatic electrochemiluminescence immunoassay analyzer (Roche Diagnostics GmbH) and its corresponding kits. Patients refrained from eating after 8 PM the evening before blood collection and fasted from 8 PM to 9 AM on the day of collection. Subcenter serum samples were transported from a 4 ℃ incubator to the main laboratory department within a 2-hour timeframe.

Thyroid ultrasound assessment: thyroid color Doppler ultrasound evaluations were conducted in the B ultrasound facility at each research center. Measurements of the width, thickness, and isthmus thickness of the left and right thyroid lobes were taken and documented.

*IL17* and *FOXP3* mRNA levels were assessed in venous blood samples collected from all patients before and after treatment. Specifically, 5 mL of venous blood was drawn into EDTA tubes, from which 250 μL was aliquoted into 1.5 mL EP tubes. Subsequently, 750 μL of Trizol was added to each tube, and the samples were stored at –80 ℃ for subsequent analysis. The quantification of *IL17* and *FOXP3* mRNA levels in peripheral blood was conducted using fluorescence quantitative PCR.

The TCM symptom score scale (see Multimedia Appendix 4 for the Symptom Score Scale) was revised for HT of liver depression and spleen deficiency type based on clinical syndrome characteristics. This revision, guided by the Beijing expert consensus on HT treatment (2021) [18], involved input from three highly experienced TCM internal medicine clinicians and preliminary investigation findings. The scale encompassed 7 items: main symptoms (fatigue, depression, irritability), secondary symptoms (abdominal distension, pain, excessive breathing, insomnia), and tongue and pulse characteristics (white or greasy fur, stringy or deep pulse). Patients were assessed for symptom severity and assigned scores, with higher scores indicating greater improvement in TCM syndromes.

The Thyroid-Specific Patient-Reported Outcome questionnaire-39-item (ThyPRO-39) scale (see Multimedia Appendix 5 for the Quality of Life Scale) is a reliable and valid instrument designed to assess the quality of life in patients with benign thyroid conditions [19]. It comprises 39 items categorized into 13 subscales, covering somatic symptoms (goiter, hyperthyroidism, hypothyroidism, and eye symptoms), physiological and psychosocial symptoms (fatigue, cognition, anxiety, depression, emotional susceptibility, social activities, daily life, and appearance), and overall quality of life. Each item is scored on a 5-point Likert scale. Subscale scores are calculated by averaging the item scores, dividing by 4, and multiplying by 100, resulting in a standard score ranging from 0 to 100. Higher scores on the ThyPRO-39 indicate a lower quality of life [20].

### Statistical Analysis Method

#### Statistical Software

For statistical analysis of data in this study, SAS 9.2 (SAS Institute) statistical software was used.

#### Fundamental Principles

In this study, all statistical inferences were made using a 2-sided test at a significance level of α=.05. The confidence intervals of the study parameters were estimated using 2-sided 95% CIs to indicate the statistical significance of differences.

#### Handling Missing Data and Sensitivity Analysis Plan for Missing Data

Missing data will be handled using Multiple Imputation under the assumption of Missing at Random. Multiple (eg, m=20) complete datasets will be generated by imputing missing values using a multivariate model incorporating key baseline and outcome variables. The planned primary and secondary analyses will be performed separately on each imputed dataset. Final estimates (eg, treatment effects, confidence intervals, and *P* values) will be obtained by pooling the results from all m datasets using Rubin rules. A sensitivity analysis assuming a Missing Not at Random scenario will be conducted to assess the robustness of the conclusions. Safety evaluations were not performed for missing data.

To ensure the robustness of the conclusions from the primary analysis, the following prespecified sensitivity analyses will be performed regarding missing data. First, the results from the primary analysis using multiple imputation will be compared with the results from an analysis based on the complete-case dataset. Second, a worst-case scenario analysis will be conducted. This analysis will assume that all participants in the intervention group with missing primary outcome data experienced treatment failure (eg, no symptomatic improvement or no reduction in antibodies), while all participants with missing data in the control group experienced treatment success. The primary outcome will be re-evaluated under this extreme assumption. These sensitivity analyses aim to assess whether the study conclusions are sensitive to the methods used for handling missing data. Consistency between the results of these analyses and the primary analysis would provide stronger support for the reliability of the final conclusions.

#### Dropout Analysis

The chi-square test was used to compare the overall dropout rate between the 2 groups. Differences in demographic characteristics, treatment received, and other variables of the dropout participants from the experimental and control groups were described and analyzed to understand the reasons for dropout.

#### Statistical Description

In this study, quantitative variables were summarized using their mean, SD, minimum, maximum, Q1, median, and Q3. Confidence intervals were provided when necessary. Quantitative variables were expressed as “mean and SD” if they followed a normal distribution and “median and IQR” if they did not follow a normal distribution. Qualitative variables are summarized in terms of frequency distribution and composition ratio.

#### Inferential Statistics

Baseline data, efficacy indicators, and safety indicators underwent inferential statistical analysis. Baseline data: statistical inferential analysis was conducted based on the type of variable to compare the comparability of the participants in the experimental and control groups. Quantitative variables were analyzed using 2-tailed *t* tests for normally distributed data and nonparametric rank-sum tests for nonnormal distribution. Qualitative variables were analyzed using chi-square tests. Efficacy indicators: comparative analysis of quantitative variables within groups was conducted using paired *t* tests for normally distributed data and paired nonparametric rank-sum tests for nonnormal distribution. Differences in efficacy indicators before and after the intervention were calculated separately for the experimental and control groups, and *t* tests or rank-sum tests were used to assess the statistical significance of these differences. Analysis of covariance, which considers the center effect, was used to provide the mean (or adjusted mean), difference in means, and 95% CIs for each group. Paired chi-square tests were used for qualitative variables, and the Wilcoxon rank-sum test was used for ranked variables. If the baseline comparability of the experimental and control groups was poor, general linear models and logistic regression models were applied for adjustment. Safety analysis: the chi-square test was used to compare the incidence of adverse events between the 2 groups, and a list was used to describe the adverse events that occurred in the trial. Intergroup and intragroup comparisons of quantitative indicators were performed using the corresponding differential tests. Laboratory test results were analyzed for changes in normal/abnormal values before and after the study, and a list was provided for abnormal values.

### Clinical Trial End

The trial will end once 140 participants have been recruited, and all participants have completed 12 weeks of treatment and 12 weeks of follow-up.

### Ethical Considerations

The research protocol and informed consent form received approval from the Clinical Research Ethics Committee of Yueyang Hospital of Integrated Traditional Chinese and Western Medicine, affiliated with Shanghai University of Traditional Chinese Medicine, on December 14, 2022 (Approval No 2022-123). Before participating in the research, we will obtain written informed consent from all participants. We will ensure voluntary participation and maximum confidentiality of the information provided during the interviews. The trial will be supervised by the Ethics Committee. Any moderate or severe adverse reactions will be reported to the Ethics Committee. Participants who experience adverse reactions during the trial will receive free access to medical care.

All research-related information will be securely stored at the research site, and all participant information will be stored in a locked filing cabinet with restricted access. To ensure participant confidentiality, all laboratory samples, reports, data collection, processes, and management forms will be identified using coded ID numbers. All records containing names or other personal information (such as informed consent forms) will be stored separately from the coded research records. All local databases will be protected using password-protected access systems. Forms, lists, logs, appointment books, and other lists linking participant identification to other identifying information will be stored in restricted access areas in individually locked files.

The relevance and necessity of the research questions, research design, and patient-centered documents (including informed consent forms, participant information sheets, symptom diaries, all follow-up forms, and promotional materials) have been subjected to review by the public. The public engagement group comprised individuals with experienced and inexperienced backgrounds. Efforts will be made to collaborate with patient advocacy groups to ensure that easily comprehensible abstracts of research findings are shared with participants and the broader patient community. The intervention burden will be comprehensively and reasonably assessed through communication between participants and researchers. While the results will not be communicated directly to the participants, the final outcomes will be disseminated. Throughout the follow-up period, all the examination results will be communicated to each participant.

All study data, including source documents, CRFs, and electronic databases, will be retained by the Sponsor for a period of at least 5 years after the completion or termination of the study. Upon expiration of the retention period, electronic data will be permanently deleted using irreversible methods, and paper records will be confidentially destroyed and incinerated. A certificate of disposal will be issued and maintained in the study files.

Blood samples collected during the study will be stored under coded identifiers at Yueyang Hospital of Integrated Traditional Chinese and Western Medicine, affiliated with Shanghai University of Traditional Chinese Medicine. For specimens authorized for future research, as permitted by the informed consent form, these specimens will be retained indefinitely in a secured biobank for use in future research projects. Any future use will be subject to approval by a Research Ethics Board. When selecting eligible participants, researchers must provide detailed information about the clinical trial, including the purpose of the trial, trial procedures, potential benefits and risks, and the rights and responsibilities of participants, so that participants can fully understand and have sufficient time to consider. After the participants’ questions have been answered satisfactorily, they will sign informed consent forms and receive the physician’s contact details so that they can contact the physician at any time if there are any changes in their condition.

### Randomization

After screening, SAS software was used to generate random numbers, and a sequential randomization plan was used to allocate the study participants to the experimental and control groups to ensure group balance. The investigator sequentially assigned a drug number to each participant based on the enrollment order and dispensed the corresponding test drug number that could not be selected arbitrarily. The number of assigned drugs remained constant throughout the study.

## Results

**Figure 1:**
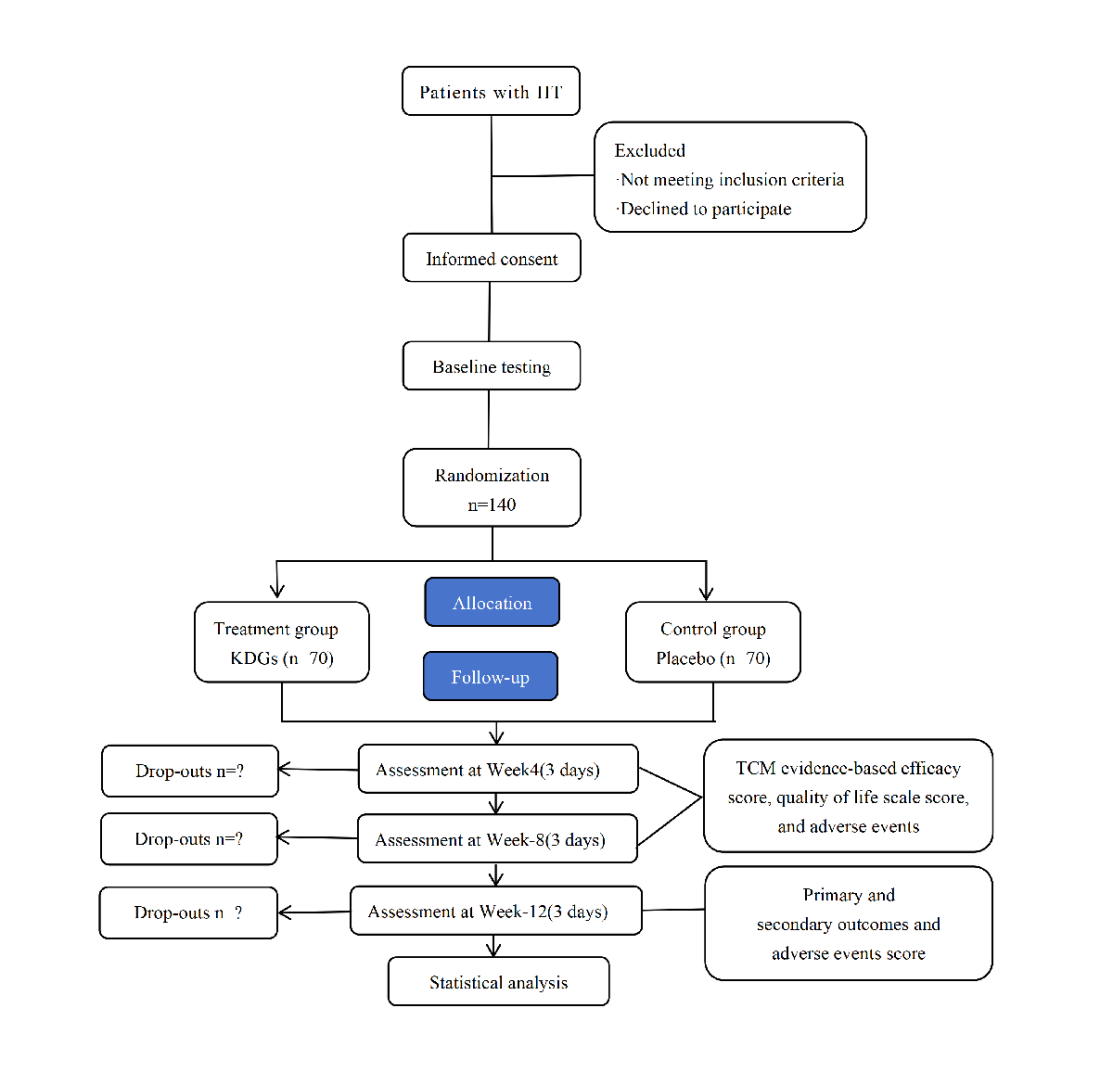
Flow chart of the study procedure. HT: Hashimoto thyroiditis; KDGs: kangjia decoction granules; TCM: traditional Chinese medicine;.

Participant recruitment commenced in June 2023. All intervention and concurrent data collection activities are scheduled for completion by May 2025, with the 12-week follow-up assessment postintervention expected to conclude by the end of May 2025. Data management has been finalized, and data analysis is presently in progress. For data inquiries, kindly reach out to the primary author. The conclusive outcomes of the research will be disseminated in a journal indexed in the Science Citation Index. The study protocol is illustrated in Figure 1.

## Discussion

### Principal Findings

This study anticipates that the KDGs can effectively reduce TPOAb and TGAb levels in patients with HT of the liver depression and spleen deficiency pattern, without significant adverse reactions. It is also expected to ameliorate patients’ clinical symptoms and signs, thereby improving their quality of life. Furthermore, KDGs may decrease the expression of *IL17* mRNA and increase the expression of *FOXP3* mRNA in patient serum, helping to improve the immune microenvironment and regulate immune balance.

During the progression of HT, aberrant immune responses and dysregulated recognition processes modulate the induction of apoptosis, leading to increased follicular cell death, irreversible fibrosis of the follicular architecture, and diminished capacity for thyroid hormone synthesis, ultimately resulting in hypothyroidism. Consequently, most patients require lifelong thyroid hormone replacement. Even with normalized thyroid hormone levels, patients often experience symptoms such as fatigue, lower limb edema, depression, and anxiety, which severely impact their quality of life. In conventional Western medicine, the primary treatment for HT relies on levothyroxine replacement to correct thyroid dysfunction. However, this approach lacks effective intervention for the early-stage immune imbalance in HT and shows limited efficacy in reducing autoantibodies (TPOAb and TGAb). Traditional Chinese Medicine identifies “liver depression and spleen deficiency” as a core pathogenesis of HT. Previous clinical observations and studies have demonstrated the efficacy of the KDGs granules in ameliorating HT symptoms and reducing autoantibody levels. KDGs is known for its properties of soothing the liver, strengthening the spleen, clearing heat, and dispersing stagnation. Animal studies have further validated its potential to lower TPOAb and TGAb levels and enhance thyroid function in model rats.

Although numerous clinical studies on Chinese medicine exist, there is a general lack of high-quality, large-sample, randomized, double-blind controlled trials. To address these gaps, this study adopts a “disease-pattern integrated” model, focusing on the core TCM pattern of “liver depression and spleen deficiency,” and uses a fixed herbal formula for intervention. Through a rigorous randomized, double-blind, controlled trial design, it aims to provide high-level evidence for TCM pattern-based treatment. Regarding efficacy evaluation, most existing studies primarily focus on changes in thyroid function and antibody titers, with insufficient exploration of the underlying mechanisms of immune imbalance [21-23]. This study innovatively incorporates key markers of Th17/Treg cell balance—the proinflammatory cytokine IL-17 and the transcription factor FOXP3—as core outcome measures. This allows for an in-depth investigation of the drug’s potential immunomodulatory targets, effectively complementing traditional evaluation models. In terms of study design, addressing limitations such as small sample sizes and lack of blinding in some previous herbal medicine research, this study uses a multicenter, randomized, double-blind, controlled trial design to minimize bias and enhance the reliability and generalizability of the findings.

This study protocol possesses several notable strengths. First, it provides a novel theoretical integration of the TCM principle of “soothing the liver and strengthening the spleen” with contemporary immunoregulatory theory. The pathophysiology of HT involves a dysregulated neuro-endocrine–immune network that manifests in systemic symptoms, a concept well-aligned with the TCM framework. We propose a mechanistic correspondence: “Liver depression” may correlate with stress-axis (hypothalamic-pituitary-adrenal axis) dysfunction and neurotransmitter disturbances underlying depression and anxiety, while “spleen deficiency” may relate to gut-barrier dysfunction, metabolic dysregulation, and inflammation-induced sarcopenia, reflecting the TCM concept of the “spleen governing the muscles.” The therapeutic principle of soothing the liver and strengthening the spleen thus represents a multitarget, systems-level intervention aimed at restoring immune-neuro–metabolic homeostasis. This integrative model offers a coherent pathophysiological rationale for targeting the co-occurrence of emotional, cognitive, and muscular symptoms in HT, underscoring the holistic perspective of TCM and providing a scientific foundation for the study hypothesis.

Second, the selection of outcome measures is comprehensive and mechanistically informed.​ Beyond assessing conventional HT biomarkers (TPOAb and TGAb), the protocol includes key mediators of the Th17/Treg pathway (*IL17* and *FOXP3* mRNA expression). This dual approach allows for a systematic evaluation​ of the intervention’s effects on both downstream autoantibody production and upstream immunologic imbalance, offering deeper insights into its potential mechanism of action.

Third, the methodology is robust. Using a multicenter, randomized, double-blind, placebo-controlled design​ minimizes bias, enhances generalizability, and is conducive to generating high-level clinical evidence, which addresses a common limitation in prior TCM interventional research.

Finally, the study addresses a clear unmet clinical need. It directly targets the persistent challenge of reducing thyroid autoantibodies and modulating the underlying immune dysregulation in HT, a gap in current conventional management. Positive results could offer a novel adjunctive strategy aimed at modifying the immune microenvironment and improving patient outcomes.

However, this study also has several limitations. First, Chinese herbal formulas are characterized by multiple components and targets. The specific active material basis of KDGs and its detailed mechanisms of action cannot be fully elucidated merely through changes in clinical indicators. Moreover, although the TCM pattern diagnosis of “liver depression and spleen deficiency” can be quantified using scales, a degree of subjectivity remains, which may affect the homogeneity of the enrolled patients regarding their pattern presentation. Second, HT is a chronic disease requiring a prolonged period for immune modulation. This study’s duration may be insufficient to fully evaluate the long-term efficacy of the intervention, the stability of antibody levels over time, and its long-term impact on disease progression (eg, changes in thyroid volume and incidence of clinical hypothyroidism). Finally, the inference of immunological changes in this study is primarily based on the detection of cytokines and transcription factors in peripheral blood, which constitutes indirect evidence. It cannot directly reflect the immune cell infiltration and inflammatory microenvironment within the thyroid gland itself, thereby limiting the depth of mechanistic interpretation.

Based on the positioning and potential findings of this study, future research directions may include, at the mechanistic level, further studies that could use flow cytometry to directly analyze the proportions of Th17 and Treg cells in patient peripheral blood. Using animal or cell models, research could delve deeper into the regulatory effects of KDGs and its active components on key signaling pathways related to Th17/Treg cell differentiation and function, such as RORγt, STAT3, and TGF-β/Smad. Furthermore, research dimensions should be expanded, for instance, by conducting long-term follow-up studies to assess the intervention’s long-term benefit in delaying progression to clinical hypothyroidism.

To facilitate translation, should the positive outcomes of this trial be confirmed, future studies could validate its efficacy and safety in real-world clinical settings. Research could also explore its synergistic effects when combined with Western medications (eg, selenium yeast and levothyroxine), aiming to establish a comprehensive management pathway for HT integrating TCM and Western medicine characteristics.

### Conclusions

This study’s findings will offer initial clinical evidence regarding the efficacy of the TCM compound KDGs in modulating peripheral immunity in patients with HT, decreasing autoimmune antibody levels, ameliorating TCM syndromes, and enhancing quality of life. These results will serve as a basis for future large-scale trial designs.

## Appendix

Multimedia Appendix 1 Reporting checklist for protocol of a clinical trial. Based on the SPIRIT 2025 guidelines.

Multimedia Appendix 2 Certificate of Analysis.

Multimedia Appendix 3 Placebo Similarity Evaluation Trial for Kangjia Decoction Granules.

Multimedia Appendix 4 Symptom Score Scale (TCM Version).

Multimedia Appendix 5 Questionnaire on quality of life related to thyroid function (ThyPRO39se).

## Abbreviations

CRF: case report form
CTCAE: Common Terminology Criteria for Adverse Events
FT3: free triiodothyronine
FT4: free thyroxine
GCP: Good Clinical Practice
HT: Hashimoto thyroiditis
IL: interleukin
KDG: kangjia decoction granule
PCR: polymerase chain reaction
REDCap: Research Electronic Data Capture
SPIRIT: Standard Protocol Items: Recommendations for Interventional Trials
T3: triiodothyronine
T4: thyroid hormone
TCM: traditional Chinese medicine
TGAb: thyroglobulin antibodies
Th17: T cell 17
ThyPRO-39: Thyroid-Specific Patient-Reported Outcome questionnaire-39-item
TPOAb: thyroid peroxidase antibody
Treg: regulatory T cell
TSH: thyroid stimulating hormone
