# Supplementary material for: Effectiveness and Safety of Kangjia Decoction Granules for the Treatment of Hashimoto Thyroiditis: Protocol for a Randomized, Double-Blinded, Placebo-Controlled, Multicenter Clinical Trial
Source: JMIR Res Protoc. 2026 Jan 26;15:e80993. doi: 10.2196/80993 (PMC12887561; doi:10.2196/80993)
Supplement: Multimedia Appendix 2 [file resprot_v15i1e80993_app2.docx]

### Multimedia Appendix 2. Symptom Score Scale (TCM Version)

| **Item** | **Criteria Score** | |
| --- | --- | --- |
| **Primary Symptoms** | | |
| **1. Fatigue** | □ 0 points: No symptoms or symptoms disappeared | □ 2 points: Mild fatigue,intolerance to labor, can perform light work |
|  | □ 4 points: Significant fatigue,barely manages daily tasks | □ 6 points: Extreme weakness, unable to perform daily activities |
| **2. Emotional Depression or Irritability** | □ 0 points: No symptoms or symptoms disappeared | □ 1 point: Occasional depression or irritability |
|  | □ 2 points: Frequent low mood or irritability | □ 3 points: Persistent depression or uncontrollable anger |
| **Secondary Symptoms** | | |
| 1. **Epigastric or**   **Hypochondriac Distension/Pain** | □ 0 points: No symptoms or symptoms disappeared | □ 2 points: Post-meal bloating, resolves within 30 minutes |
|  | □ 4 points: Daily distension/pain for <2 hours | □ 6 points: Persistent distension/pain requiring analgesics |
| **4. Frequent Sighing** | □ 0 points: No symptoms or symptoms disappeared | □ 1 point: Occasional sighing |
|  | □ 2 points: Sighing triggered by stress | □ 3 points: Frequent sighing |
| **5. Insomnia** | □ 0 points: No symptoms or symptoms disappeared | □ 1 point: Light sleep/early awakening,no impact on work |
|  | □ 2 points: Sleep <4 hours/day, but can work | □ 3 points: Severe insomnia, unable to work |
| **Tongue & Pulse** | | |
| **6. Tongue Coating** | □ 1 point: White/greasy coating | □ 0 points: Other |
| **7. Pulse Condition** | □ 1 point: Wiry or thin pulse | □ 0 points: Other |
| **Total Score** |  | |
