# Supplementary material for: Effectiveness and Safety of Kangjia Decoction Granules for the Treatment of Hashimoto Thyroiditis: Protocol for a Randomized, Double-Blinded, Placebo-Controlled, Multicenter Clinical Trial
Source: JMIR Res Protoc. 2026 Jan 26;15:e80993. doi: 10.2196/80993 (PMC12887561; doi:10.2196/80993)
Supplement: Multimedia Appendix 4 [file resprot_v15i1e80993_app4.docx]

**Multimedia Appendix 4.** Certificate of Pharmaceutical Preparation Analysis

**CERTIFICATE OF ANALYSIS**

**Certificate No.: 23W-004 Date of Issue: 05-May-2023**

| **Product Information:** |  |
| --- | --- |
| Product Name: Kangjiafang Granules | Batch No.: 20230401 |
| Dosage Form: Granules | Batch Size: 52106 packs |
| Strength: 5 g per pack | Manufacturing Date: 01-Apr-2023 |
| Expiry Date: 01-Apr-2026 | Specification: SP-EP-R004-00 |

TEST RESULTS

| **Test Parameter** | **Specification** | **Conclusion** |
| --- | --- | --- |
| **Description** | Pale brown to brown granules; Odour, slightly aromatic; taste, sweet and slightly bitter. | Complies |
| **Identification**  （1） | A brown spot in the chromatogram obtained with the test  solution corresponds in position and colour to the spot in the  chromatogram obtained with Astragali Radix reference solution and astragaloside IV reference solution. Examine under  ultraviolet ' light at 365 nm, an orange-yellow fluorescent spot in the chromatogram obtained with the test solution corresponds in position and colour to the spot obtained with astragaloside IV  reference solution and Astragali Radix reference solution. | Complies |
| （2） | The fluorescent band in the choromatogram obtained with the  test solution corresponds in position and colour to the band in the | Complies |

| **Test Parameter** | **Specification** | **Conclusion** |
| --- | --- | --- |
| （3） | chromatogram obtained with Prunellae Spica reference solution. The fluorescent spot in the choromatogram obtained with the  test solution corresponds in position and colour to the spot in the chromatogram obtained with Atractylodis Macrocephalae  Rhizoma reference solution. | Complies |
| （4） | The spot in the chromatogram obtained with the test solution corresponds in position and colour to the spot in the  chromatogram obtained with Forsythiae Fructus reference solution and forsythin reference solution. | Complies |
| **Tests**  Water  Particle Size | Not more than 8.0 per cent  It contains not more than 15% of exceptive particles, calculated as the total amount of particles pass through No. 5 sieve and not pass No. 1 sieve. | 6.0 %  3.0 % |
| Weight Variation Solubility | The limit should be within ±7%  Dissolve or slight cloudiness, no foreign matter or charred particles are permitted. | -4.6 %~+2.4 % Complies |
| **Microbial limit**  Total Aerobic  Microbial count  Total combined yeast and mold counts  Escherichia coli  **Assay**  Astragali Radix | Not more than 103 CFU/g  Not more than 102 CFU/g  Absent  It contents not less than 0. 17 mg ofastragaloside IV（C22H22O10） per pack, referred to Astragali Radix. | 300 CFU/g  70 CFU/g  Complies  **Complies**  1.08 mg per pack |
| Prunellae Spica | It contents not less than 3. 1 mg of rosmarinic acid （C18H16O8） per pack, referred to Prunella Spica. | 8.06 mg per pack |

**Conclusion:**

The results above indicate that Batch No. 20230401 complies with all the specifications.

APPROVED BY:


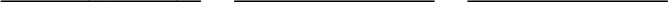


(Signature) (Printed Name) (Date)

Title: Quality Assurance Manager
