# Supplementary material for: Effectiveness and Safety of Kangjia Decoction Granules for the Treatment of Hashimoto Thyroiditis: Protocol for a Randomized, Double-Blinded, Placebo-Controlled, Multicenter Clinical Trial
Source: JMIR Res Protoc. 2026 Jan 26;15:e80993. doi: 10.2196/80993 (PMC12887561; doi:10.2196/80993)
Supplement: Multimedia Appendix 5 [file resprot_v15i1e80993_app5.docx]

**Multimedia Appendix 5.** Placebo Similarity Evaluation Trial for Kangjia Decoction Granules

**1. Trial Objective**

To evaluate the similarity of the placebo using the artificial scoring method.

**2. Trial Subjects**

(1) Investigational Product: KangJiafang Granules (20230401)

(2) Placebo: KangJiafang Granules Placebo (20230401)

**3. Trial Content**

**(1) Evaluation Method:**

The placebo and the investigational product are distributed simultaneously to each evaluator. Evaluators are required to score the similarity of the two products from four aspects: Appearance (Clarity), Color, Odor, and Taste. Scores range from 0 to 10. The meaning of each score range is shown in the table below.

| Score Range | 0-1 | 2-3 | 4-6 | 7-8 | 9-10 |
| --- | --- | --- | --- | --- | --- |
| Meaning | Completely  Different | Quite  Different | Uncertain | Quite Similar | Identical |

The products tasted by the evaluators are both prepared by the trial personnel according to the instructions for use (i.e., dissolved in liquid).

**(2) Evaluator Selection:Number of evaluators shall be no less than 10.**

**(3) Similarity Score Sheet:**

**Placebo Similarity Score Sheet**

**Please rinse your mouth after tasting each sample.**

| **Sensory Level** | | **Completely**  **Different** | | **Quite**  **Different** | | **Uncertain** | | | **Quite**  **Similar** | | **Identical** | |
| --- | --- | --- | --- | --- | --- | --- | --- | --- | --- | --- | --- | --- |
| **Score** | | **0** | **1** | **2** | **3** | **4** | **5** | **6** | **7** | **8** | **9** | **10** |
| **Item** | **Clarity** |  |  |  |  |  |  |  |  |  |  |  |
|  | **Odor** |  |  |  |  |  |  |  |  |  |  |  |
|  | **Color** |  |  |  |  |  |  |  |  |  |  |  |
|  | **Taste** |  |  |  |  |  |  |  |  |  |  |  |

**Signature: _______________________ Date: ________________**

**4. Trial Result**

**(1) Record**

| **No.** | **Clarity** | **Odor** | **Color** | **Taste** |
| --- | --- | --- | --- | --- |
| Subject 1 | 6 | 8 | 7 | 5 |
| Subject 2 | 9 | 7 | 9 | 5 |
| Subject 3 | 8 | 7 | 9 | 3 |
| Subject 4 | 9 | 7 | 8 | 5 |
| Subject 5 | 7 | 8 | 10 | 4 |
| Subject 6 | 8 | 5 | 10 | 5 |
| Subject 7 | 6 | 7 | 6 | 9 |
| Subject 8 | 8 | 6 | 9 | 7 |
| Subject 9 | 10 | 9 | 7 | 9 |
| Subject 10 | 8 | 7 | 8 | 6 |

**(2) Calculation**

|  | **Clarity** | **Odor** | **Color** | **Taste** |
| --- | --- | --- | --- | --- |
| **Mean** | 7.9 | 7. 1 | 8.3 | 5.8 |
| **Median** | 8 | 7 | 8 | 5 |

Evaluation results: The mean and median scores indicate high similarity in

clarity, color, and odor, while the similarity score for taste is relatively low.

**(3) Analysis**

|  | **Clarity** | | **Odor** | | **Color** | | **Taste** | |
| --- | --- | --- | --- | --- | --- | --- | --- | --- |
|  | **quantity** | **similarity** | **quantity** | **similarity** | **quantity** | **similarity** | **quantity** | **similarity** |
| **Similar count** | **10** | **100%** | **9** | **90%** | **10** | **100%** | **4** | **40%** |
| **Dissimilar count** | **0** |  | **1** |  | **0** |  | **6** |  |

**In summary, the evaluation indicates a high degree of similarity in terms of clarity, odor, and color, whereas the taste similarity is significantly lower.**
